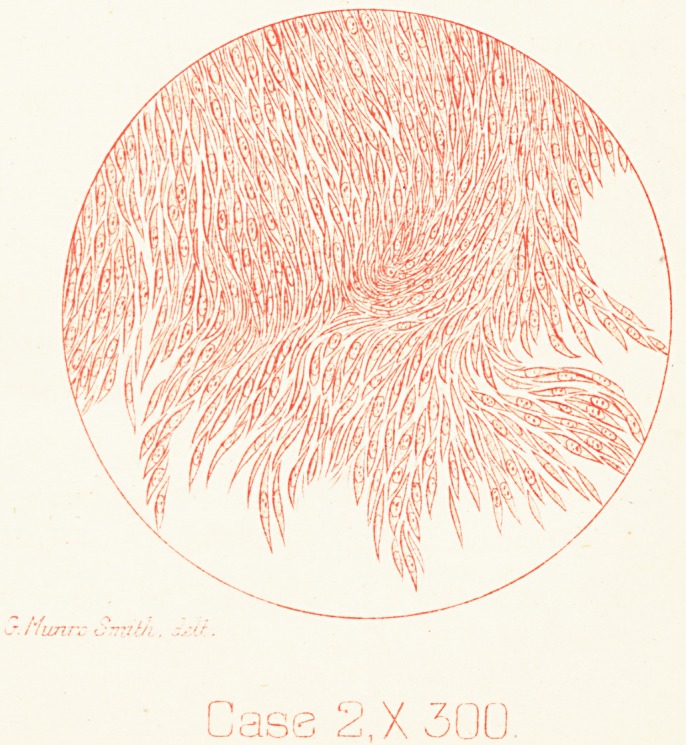# Two Cases of Compression of the Spinal Cord by Sarcomatous Growths from the Soft Membranes

**Published:** 1883-07

**Authors:** E. Long Fox

**Affiliations:** Consulting Physician to the Bristol Royal Infirmary


### Clinical Records.

---

TWO CASES OF COMPRESSION OF THE SPINAL CORD BY SARCOMATOUS GROWTHS FROM THE SOFT MEMBRANES. By E. LONG FOX, M.D. Oxon., F.R.C.P., Consulting Physician to the Bristol Royal Infirmary.

THE following case is recorded as an instance of a rare lesion, both as to pathology and position. The case was under the care of Dr. Colman, by whose permission it is reproduced here.

Mrs. G., 35. In the early part of 1882 she was lifting a heavy window, and felt it too much for her, and was sick. About one month after this she began to feel pain in the right arm, and some weakness of the right hand. These symptoms gradually increased for several months. In the summer of the same year she got wet through. Shortly after this, loss of motor power affected both arms and both legs.

When seen in consultation in November, her state was as follows :—

Almost complete motor paralysis of both arms. She can move each hand about a quarter of an inch if she moves both hands at once. Sensation in hands and arms much impaired but not absent. In the legs, motor paralysis was complete, and sensation dull, but better than in the arms.

Some weeks ago the urine passed involuntarily; now no incontinence of urine, but some difficulty in commencing micturition. Some difficulty also in expelling fæces; constipation throughout the illness. Pupils regular.

Knee jerk exaggerated in both legs. Well marked ancle-clonus on both sides. Tapping over the shin-bone causes ancle-clonus. Tapping over the lower part of biceps causes contraction of little and ring finger, and this on each side. Plantar reflexes rather exaggerated. No marked atrophy anywhere, except that the muscles of the arms and hands are small and flaccid from disuse, especially on the left side. No bed sore. She looks bright and rosy and well in the face. Can be placed in a chair in a semi-erect position. The extremities are often cold, or she is cold on one side of the body and hot on the other. No other vasomotor phenomenon. There is tenderness and perhaps a little swelling over the sixth cervical vertebra, over which she has had a seton for six weeks. She cannot turn her head quite well. No ocular disturbances. Brain clear. She sleeps and eats fairly. The diagnosis then made was that of a minute hæmorrhage or inflammatory exudation, beginning on the right spinal membranes about the sixth cervical vertebra. It was thought that the inflammatory action had spread round to the other side—that the original exudation had probably developed into a growth compressing the cord on the anterior surface, causing compression-myelitis; but that the anterior cornua has not yet suffered much; and that descending lateral sclerosis supervened as a secondary result. It was probable also that the original lesion had been caused by the strain at the window.

Her health remained tolerably good during the winter, except that for some months she suffered from severe carbuncles near the seat of lesion. She went out during the winter, but caught cold in the early part of April. When seen in consultation on April 13th, it was found that she had very slight bronchial catarrh, and that she was quite unable to cough. There was no intercostal movement whatever, the respiration being wholly diaphragmatic. There was a central bed sore. She lived in this state for six days. The intellect was clear until within an hour of death.

*Post Mortem Examination.*—The spinal canal alone examined. On removing the cord and its membranes from the canal the cervical bulb was evidently abnormally large. The dura mater healthy on both surfaces. The arachnoid was thickened, so much so as to conceal the subjacent parts. A hard, rather vascular tumour, about an inch and a half long and three-quarters of an inch broad, lay on the anterior surface of the cord, destroying the cord by pressure at this spot, except apparently the posterior columns (Pl. III.). All the cord below the tumour down to the lumbar region was somewhat softened; at the level of the growth it was almost deliquescent. The shape and position of the growth and of the cord itself is seen in the accompanying plate. The arachnoid was firmly adherent to the tumour. At the upper end of the tumour, exactly at the lateral surface, the arachnoid was adherent to the dura mater, as though at this point the pressure of the growth had been exerted outwards, and a minute region of inflammation of both arachnoid layers had been the result. The tumour could be easily detached from the cord and turned back; a thin pia mater lay between it and the cord. The growth was a spindle-celled sarcoma (Pl. IV., Case 1), but more fibrous than Case 2 about to be mentioned.

The chief points in the case are—

1. The position of the lesion. The pressure being from the anterior towards the posterior portion of the cord explains the motor paralysis being so much more

Plate III.

Sarcomatous Growth compressing the Spinal Cord.

intense and advanced than the sensory. The lesion not reaching as high as the fourth cervical nerve necessitates the freedom of the phrenics, and thus the normal action of the diaphragm.

2. Life was prolonged for six days after all thoracic respiration had ceased. This is unusually long compared with the cases of cervical compression from accident, where life seldom persists for more than four days with diaphragmatic breathing only.

3. The almost complete absence of vasomotor and oculo-pupillary phenomena is striking. Their absence in such extensive lesion of the cord in this region points to the possibility of independent action of the cervical sympathetic ganglia.

4. Was there any connection between the lesion found and the accident from which the phenomena dated? In a case recorded in the Bristol Infirmary Reports by Dr. Shingleton Smith and myself, the lesion, which seemed to date from a similar slight accident, did not lead to fatal results for five years. But in this case, as indeed is much more usual in cervical paraplegia, the lesion affected the dura mater. Was the more rapid course in Dr. Colman's case due to the arachnoid being the membrane affected? Or had the disease been going on for a considerable period in an early stage, and only developed these acute phenomena in consequence of the strain?

5. From the position of the tumours one would hardly have expected that the motor paralysis of all the muscles of the arm should have been so complete. There was no difference in this respect between the deltoid and the muscles of the upper arm and those of the hand. Judging from the experiments of Drs. Ferrier and Yeo, the muscles of the upper arm might have escaped for a longer period.

6. As to the nature of the tumour. Speaking generally, compression-myelitis from morbid growths will occur either from enchondroma, exostosis, or carcinoma growing from the bones themselves, and with a very slow course, even sometimes from hydatids, or more commonly from disease of the membranes, sarcoma and carcinoma, with their varieties, glioma, friboma and myxoma, the latter especially from the soft membranes. The majority of these have their starting-point on the inner side of the dura mater. Of these, carcinoma and myxoma are apt to extend largely, whilst sarcoma and melano-sarcoma have generally smaller boundaries. Lipoma, tubercle, and gumma may also own the membranes as their origin. Or, again, the cord itself may be the seat of carcinoma, sarcoma, glioma, and myxoma, and more rarely of syphilitic gumma and tubercle.

In the case recorded the visceral surface of the arachnoid was evidently the original starting-point of the growth. The tumour seemed vascular, even to the naked eye, so much so at one spot in the centre of its anterior surface as to suggest the idea of melano-sarcoma.

In connection with this case may be mentioned another that died in the Infirmary some years ago. Apart from the rarity of such cases it is interesting as affording an illustration of the union of lepto-meningitis with tumour of the soft membranes.

*Case* 2.—John D., 29. Has never been a robust man, has had cough and dyspnœa at night occasionally; his mother, grandmother, and sister died phthisical. No history of nerve disease, except that most of the family were affected with melancholia. No syphilis, always temperate, and a total abstainer for four years. Three months ago he was in good health. Shortly after this he began to experience shooting pains in the arms, much worse when he was lying down, especially at night. No numbness, no spasm. The fingers of the right hand gradually became flexed, and the power of extending them gone. Shortly after this the fingers of the left hand became similarly affected. About five weeks previously he felt the toes of the right foot becoming numb, with a slight pricking sensation; this feeling of numbness gradually extended upwards. A day or two after the right foot began to be thus affected the left foot was seized in exactly a similar manner. The numbness has continued to progress upwards. When it reached the calves of the legs the patient began to have the girdle feeling round his chest, and also to find slight difficulty in passing his water, especially at night. He had to have a catheter passed occasionally. Frequent priapism. No pain in the back on percussing vertebræ. Special senses good, except that his sight was not good, and that sensation was dull in certain regions. No sensation in legs, thighs, abdomen, and chest as high as the third rib, on admission. No hyperæsthesia anywhere. Plantar reflexes normal. Deep reflexes not understood at that time. No voluntary power in the lower extremities. He can move his arms over his head, but cannot use the fingers at all from extreme flexion. He was unable to feed himself. Respirations 24 per minute, thoracic and diaphragmatic. He lived two months after admission, the loss of power and anæsthesia rather increasing in intensity, though not extending higher. His legs were continually jerking, and at times were completely drawn up, he being unconscious of the fact unless he was looking at them. The muscles of the upper extremity became at last completely paralysed, except that the trapezius and deltoid retained a small amount of power. Thoracic breathing diminished toward the close of life.

*Post Mortem Examination.*—On opening the spinal cavity the dura mater seemed unusually full and tense. No morbid appearances otherwise. No adhesions within the cavity. The cord, or what at first sight appeared to be the cord, seemed to be generally larger and fuller than usual; and at its upper portion, midway between the cervical bulb and second cervical vertebra, was a swelling on its left posterior aspect. On section this was found to be a tumour, with well-defined margin of purplish colour, tolerably firm to touch, an inch and a half in length and three-quarters of an inch in diameter. Much softening of the cord round the tumour, and half an inch above and below it. On making a transverse section of the cord, it was found that the cord proper was smaller than normal, but the great increase of size that was visible on opening the dura mater was owing to a belt of yellowish-grey substance, fully a quarter of an inch in thickness, which enveloped the cord equally on all sides. It was not quite so abundant in the immediate region of the tumour, but elsewhere it existed, as above described, from the highest portion of the cord to the cauda equina. It could readily be separated from the cord. The pia mater and arachnoid could not be separated from it. The brain itself was healthy, except that at the base the pia mater covering the pons and around the medulla oblongata was very much thickened, and studded with what appeared to be rounded masses of exudation, extending along the vessels as far as the sylvian fissure. No brain tissue came away on stripping off the pia mater. Microscopically the spinal tumour was seen to be a spindle-celled sarcoma (Pl. IV., Case 2).

Plate IV

Case 1, X 300.

G. Munro Smith, del.

Case 2, X 300.

## Figures and Tables

**Figure f1:**
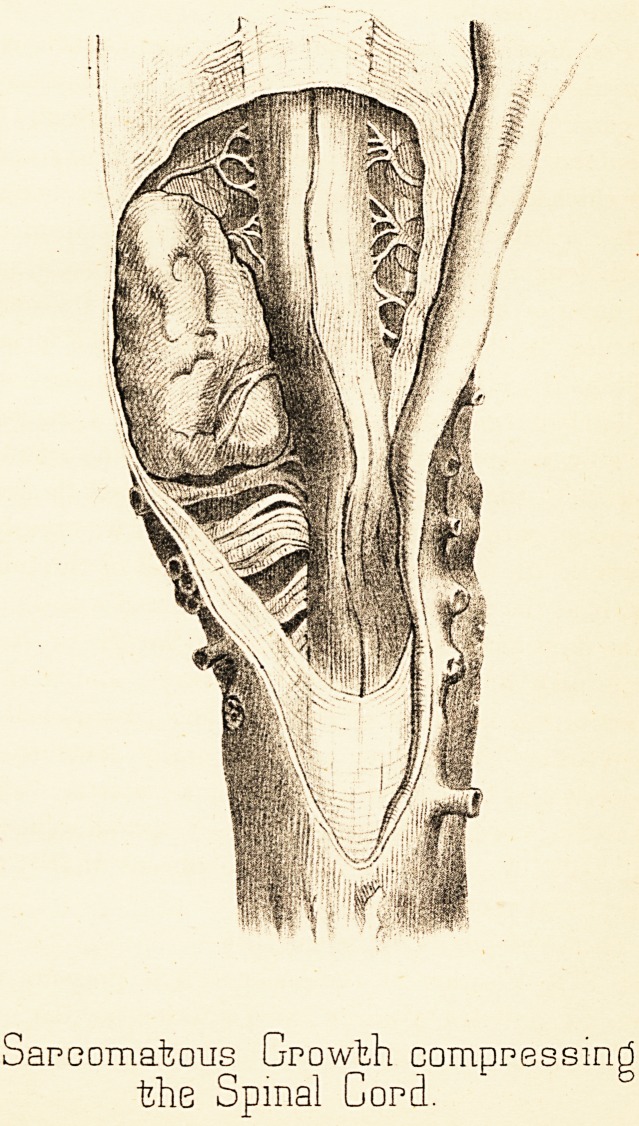


**Figure f2:**
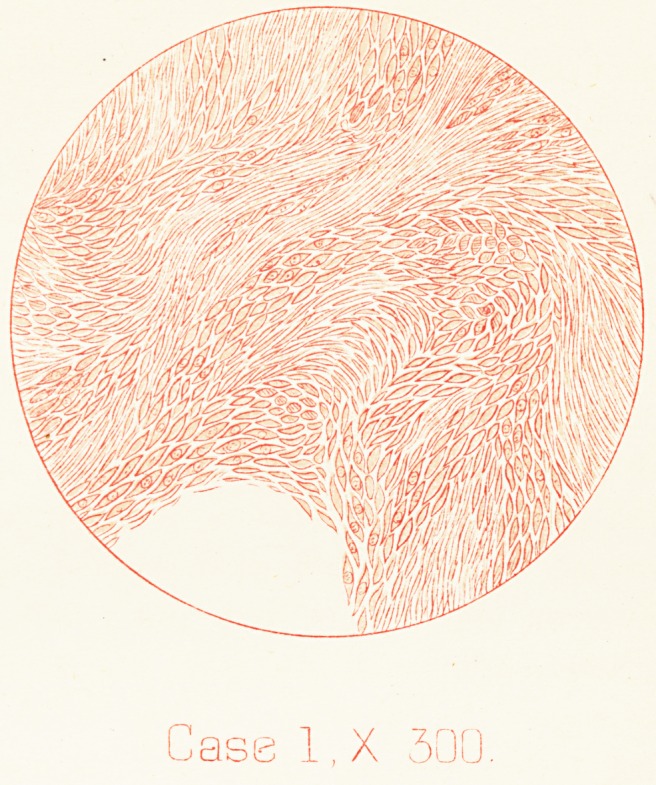


**Figure f3:**